# Supplementary figures and images for: Plasmodium NEK1 coordinates MTOC organisation and kinetochore attachment during rapid mitosis in male gamete formation
Source: PLoS Biol. 2024 Sep 10;22(9):e3002802. doi: 10.1371/journal.pbio.3002802 (PMC11414920; doi:10.1371/journal.pbio.3002802)

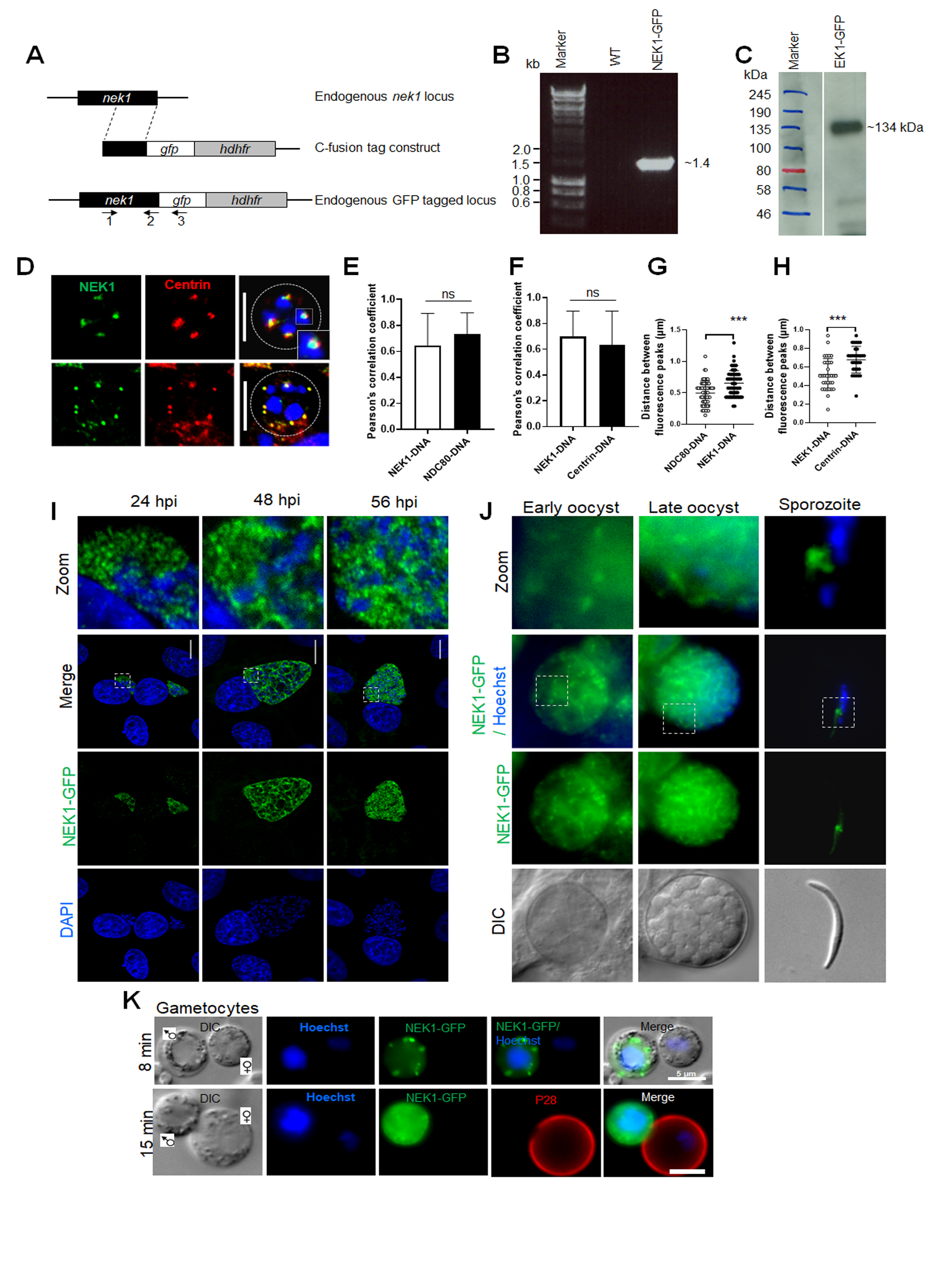

Supplement: S1 Fig — (A) Schematic representation of the endogenous Pbnek1 locus, the GFP-tagging construct, and the recombined nek1 locus following single homologous recombination. Arrows 1 and 3 indicate the position of PCR primers used to confirm successful integration of the construct. (B) Diagnostic PCR of nek1 and WT parasites using primers IntNk1tg (Arrow 1) and ol492 (Arrow 3) to show the correct integration. Integration of the nek1 tagging construct gives a band of 1,400 bp. (C) Western blot showing the expression of endogenous NEK1 detected by ant-GFP antibody. (D) Indirect immunofluorescence assays (IFAs) with centrin- and GFP-specific antibodies showing location of NEK1–GFP (green) in relation to centrin (red) and DNA (Hoechst, blue). Merge: green, red, and blue images merged; inset shows higher magnification of nucleus in box in main panel. Guide: schematic of NEK1, Centrin and DNA location at 3 stages of schizogony. The dotted line indicates the periphery of the cell. More than 20 images were analysed in more than 3 different experiments for each time point; the scale bar is 5 μm. (E) The bar diagram shows the Pearson’s correlation coefficient values showing the overlap of DNA with NEK1 and NDC80. More than 30 images were analysed in more than 3 different experiments for each time point. (F) The bar diagram shows the Pearson’s correlation coefficient values showing the overlap of DNA with NEK1 and Centrin. More than 30 images were analysed in more than 3 different experiments for each time point. ns = nonsignificant. (G) Dot plot showing individual data points, means, and standard deviation of fluorescence intensity profiles for DNA, NEK1, and centrin-4. More than 30 focal points were analysed. ***P > 0.001. (H) Dot plot showing individual data points, means, and standard deviation of fluorescence intensity profiles for DNA, NEK1, and NDC80. More than 30 focal points were analysed. ***P > 0.001. (I) Live cell images showing the location of NEK1-GFP in liver stages. More th [file pbio.3002802.s001.tif]

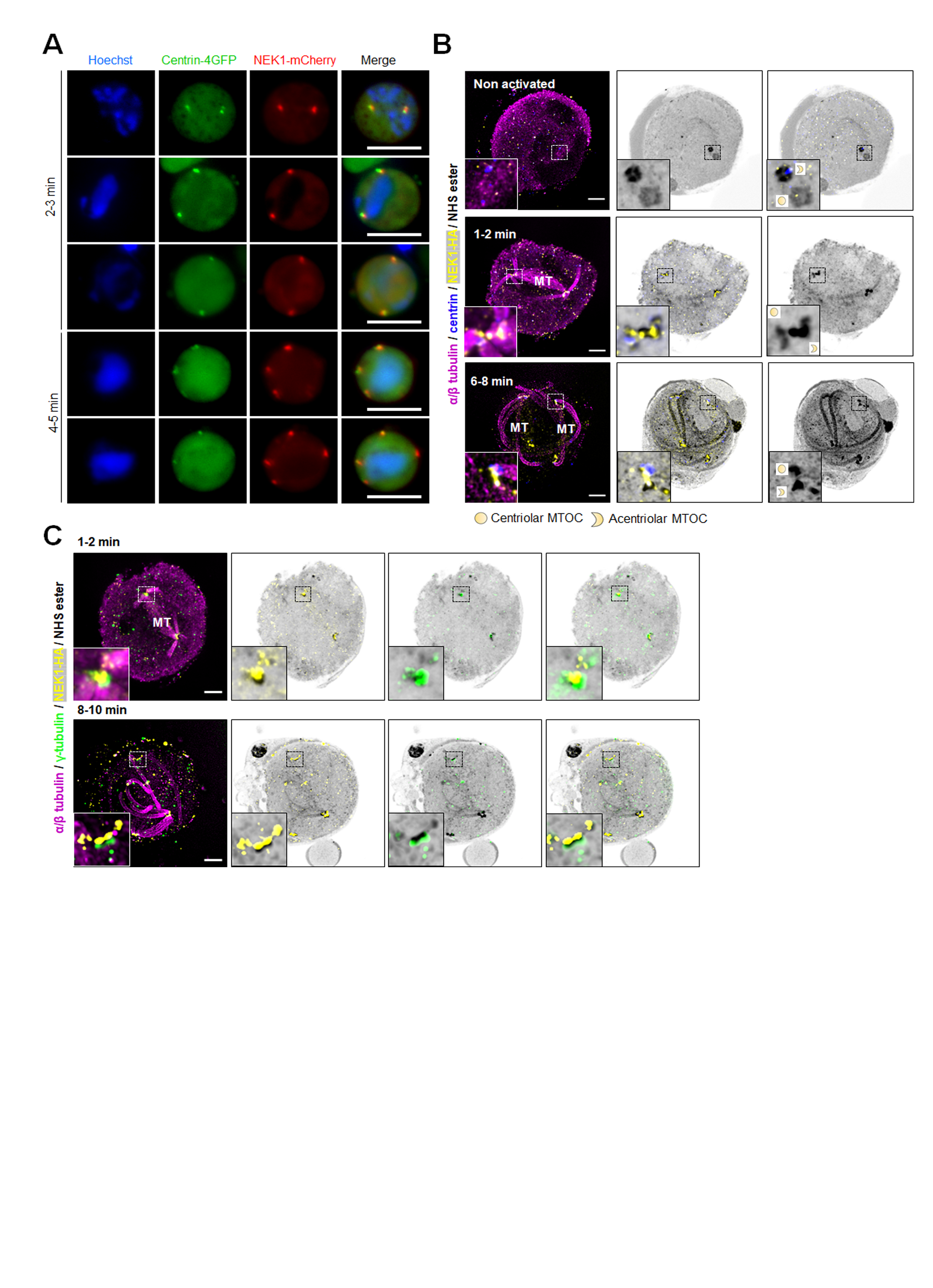

Supplement: S2 Fig — (A) Live cell imaging showing the location of NEK1-mCherry (red) in relation to centrin-4-GFP (green) in male gametocytes at different time points after activation. Scale bar = 5 μm. (B) Expansion microscopy showing location of NEK1-HA (yellow), centrin (blue), and α/β tubulin (magenta) detected with specific antibodies. Amine reactivity with NHS-ester is shown in shades of grey. The boxed areas in panels correspond to the zoomed insets. Axonemes: A; cMTOC: centriolar MTOC; aMTOC: acentriolar MTOC; spindle microtubules: MT. The guide illustrates NEK1 location relative to the MTOC. More than 20 images were analysed in 3 different experiments for each time point. Scale bar = 5 μm. (C) Expansion microscopy showing location of NEK1-HA (yellow), γ-tubulin (green), and α/β tubulin (magenta) stained with antibodies. Amine reactivity with NHS-ester is shown in shades of grey. The boxed areas in panels correspond to the zoomed insets. Axonemes: A; spindle microtubules: MT. More than 20 images were analysed in 3 different experiments. Scale bar = 5 μm. (TIF) [file pbio.3002802.s002.tif]

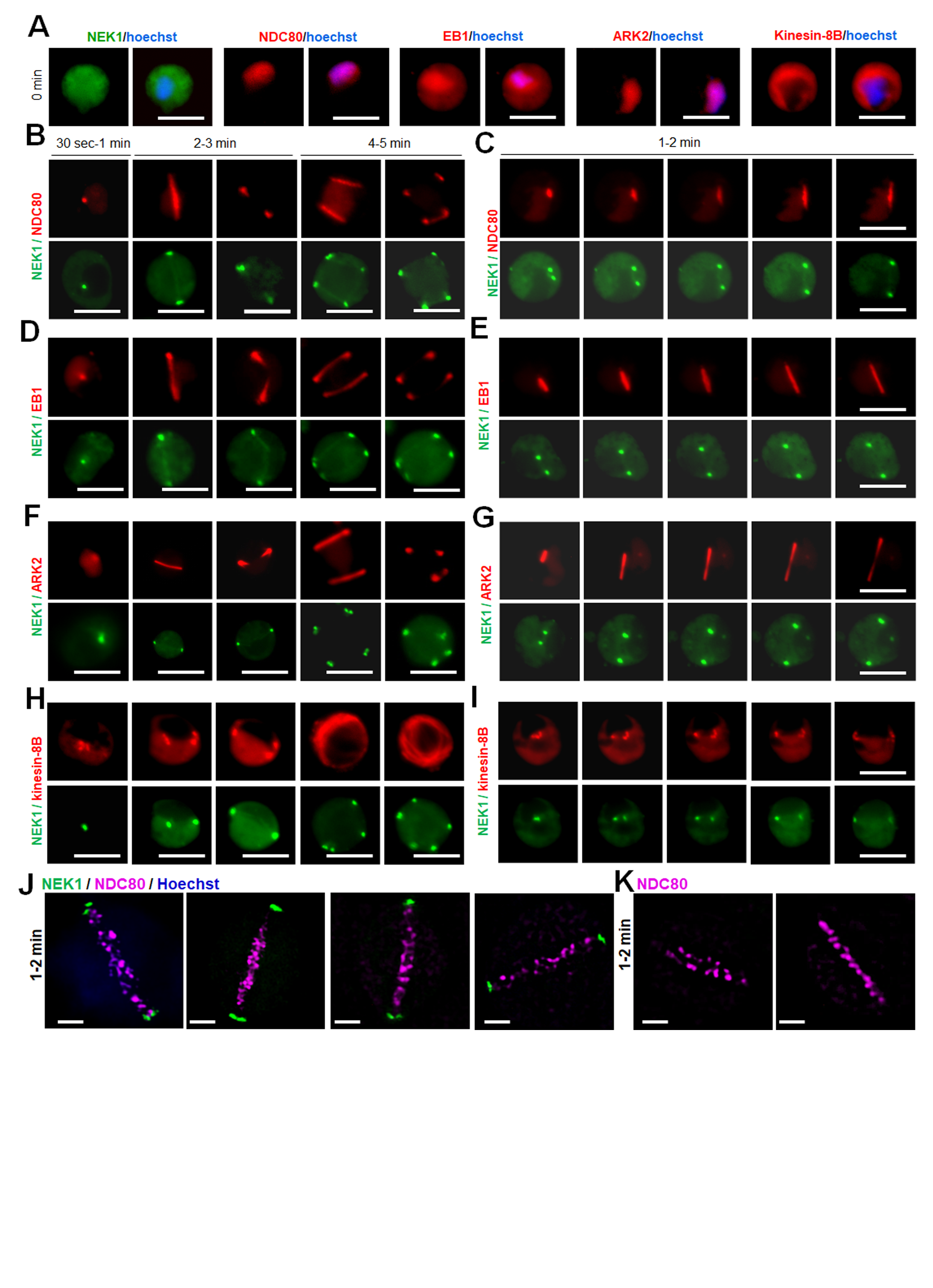

Supplement: S3 Fig — (A) Live cell imaging showing the location of NEK1-GFP, NDC80-mCherry, EB1-mCherry, ARK2-mCherry, and kinesin-8B-mCherry before activation of male gametocytes. (B) Live cell imaging showing the dynamics of NEK1-GFP (green) in relation to kinetochore marker (NDC80-mCherry [red]) at different time points during gametogenesis. (C) Stills from time lapse imaging showing the dynamics of NEK1-GFP (green) every 5 s in relation to kinetochore/spindle marker (NDC80-mCherry [red]) after 1 to 2 min activation. (D) Live cell imaging showing the dynamics of NEK1-GFP (green) in relation to spindle marker (EB1-mCherry [red]) at different time points during gametogenesis. (E) Stills from time lapse imaging showing the dynamics of NEK1-GFP (green) every 5 s in relation to spindle marker (EB1-mCherry [red]) after 1 to 2 min activation. (F) Live cell imaging showing the dynamics of NEK1-GFP (green) in relation to spindle associated marker (ARK2-mCherry [red]) at different time points during gametogenesis. (G) Stills from time lapse imaging showing the dynamics of NEK1-GFP (green) every 5 s in relation to spindle associated marker (ARK2-mCherry [red]) after 1 to 2 min activation. (H) Live cell imaging showing location of NEK1-GFP (green) in relation to the basal body and axoneme marker, kinesin-8B-mCherry (red) at different time points during gametogenesis. (I) Stills from time lapse imaging showing the dynamics of NEK1-GFP (green) every 5 s in relation to kinesin-8B-mCherry (red) after 1 to 3 min activation. Scale bar = 5 μm. (J) Super-resolution 3D imaging of NEK1-GFP and NDC80-mCherry in gametocytes fixed at 1 to 2 min post activation. The focal points of NDC80 represent kinetochores. Scale bar = 1 μm. (K) Super-resolution 3D imaging of NDC80-mCherry in gametocytes fixed at 1 to 2 min post activation. Scale bar = 1 μm. (TIF) [file pbio.3002802.s003.tif]

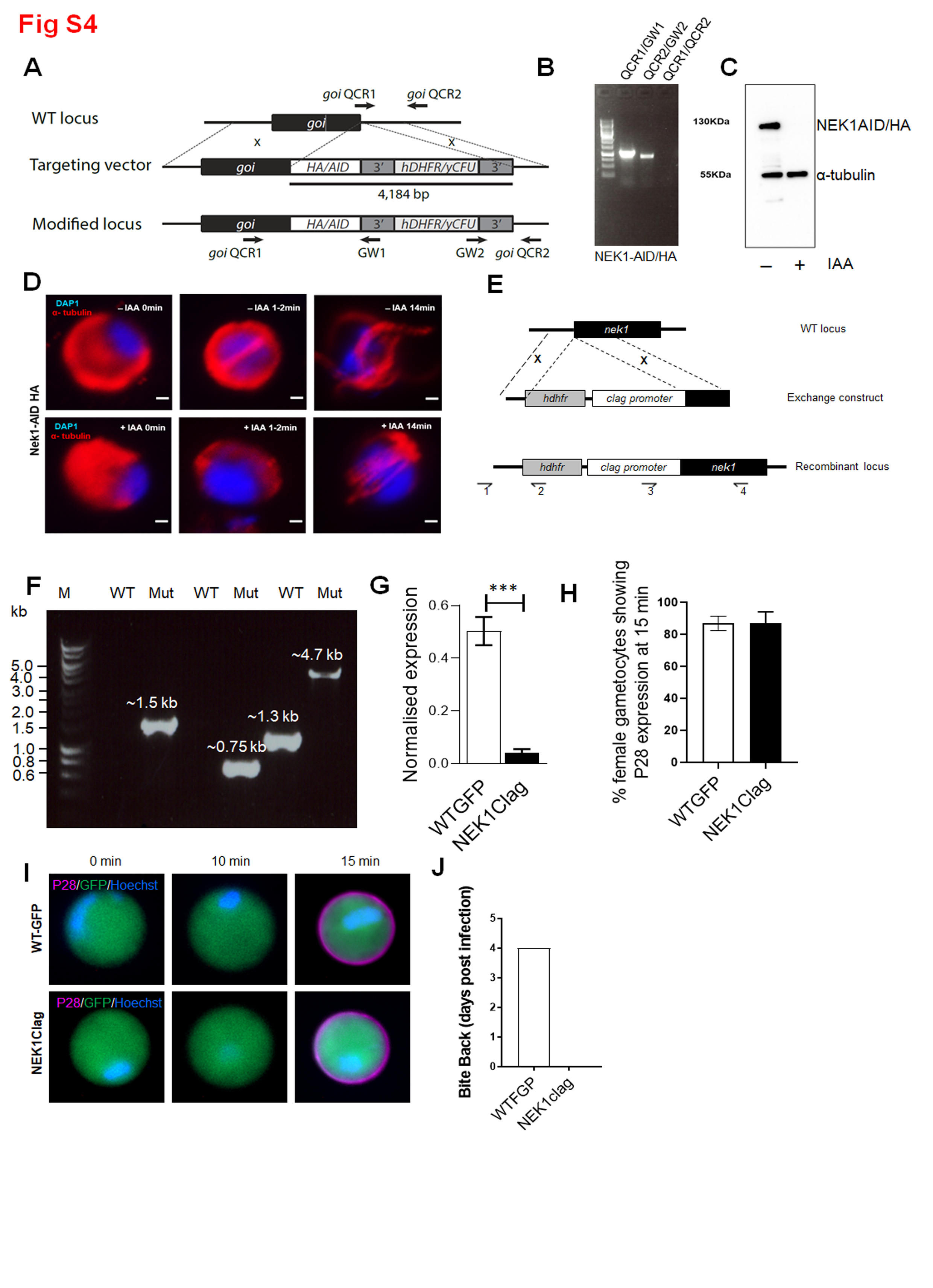

Supplement: S4 Fig — (A) Schematic representation of auxin inducible degron (AID) strategy to generate NEK1-AID/HA parasites. goi; gene of interest (nek1). (B) Integration PCR of the NEK1-AID/HA construct in the ark2 locus. Oligonucleotides used for PCR genotyping are indicated, and agarose gels to analyse the corresponding PCR products from genotyping reactions are shown. (C) NEK1-AID/HA protein expression level as measured by western blotting using anti-HA antibodies upon addition of auxin to mature purified gametocytes; α-tubulin served as a loading control. (D) Immunofluorescence images of male gametocytes showing tubulin staining at different time points. DNA is stained with DAPI (blue). More than 20 images were analysed in 3 different experiments for each time point. Scale bar = 1 μm. (E) Schematic representation of the promoter swap strategy to construct NEK1clag parasites (placing NEK1 under the control of the clag promoter) by double homologous recombination. Arrows 1 and 2 indicate the primer positions used to confirm 5′ integration and arrows 3 and 4 indicate the primers used to confirm 3′ integration. (F) Integration PCR of the promotor swap construct into the NEK1 locus. Primer 1 (IntPTD14_5) and primer 2 (5′-IntPTD) were used to confirm successful integration of the selectable marker, resulting in a band of approximately 1,500 bp. Primer 3 (3′-intPTclag) and primer 4 (IntPTD14_3) were used to determine the successful integration of the clag promoter, resulting in a band of approximately 750 bp. Primer 1 (IntPTD14_5) and primer 4 (IntPTD14_3) were used to confirm a complete knock-in of the construct with a band at approximately 4.7 kb and the absence of a band at approximately 1.3 kb. Representative image of more than 3 experiments. (G) qRT-PCR showing normalised expression of NEK1 transcripts in NEK1clag and WT-GFP parasites. Shown is mean ± SEM; n = 3 independent experiments. Student t test was used to examine significant difference. ***p < 0.01. (H) Gametogenesis in fema [file pbio.3002802.s004.tif]

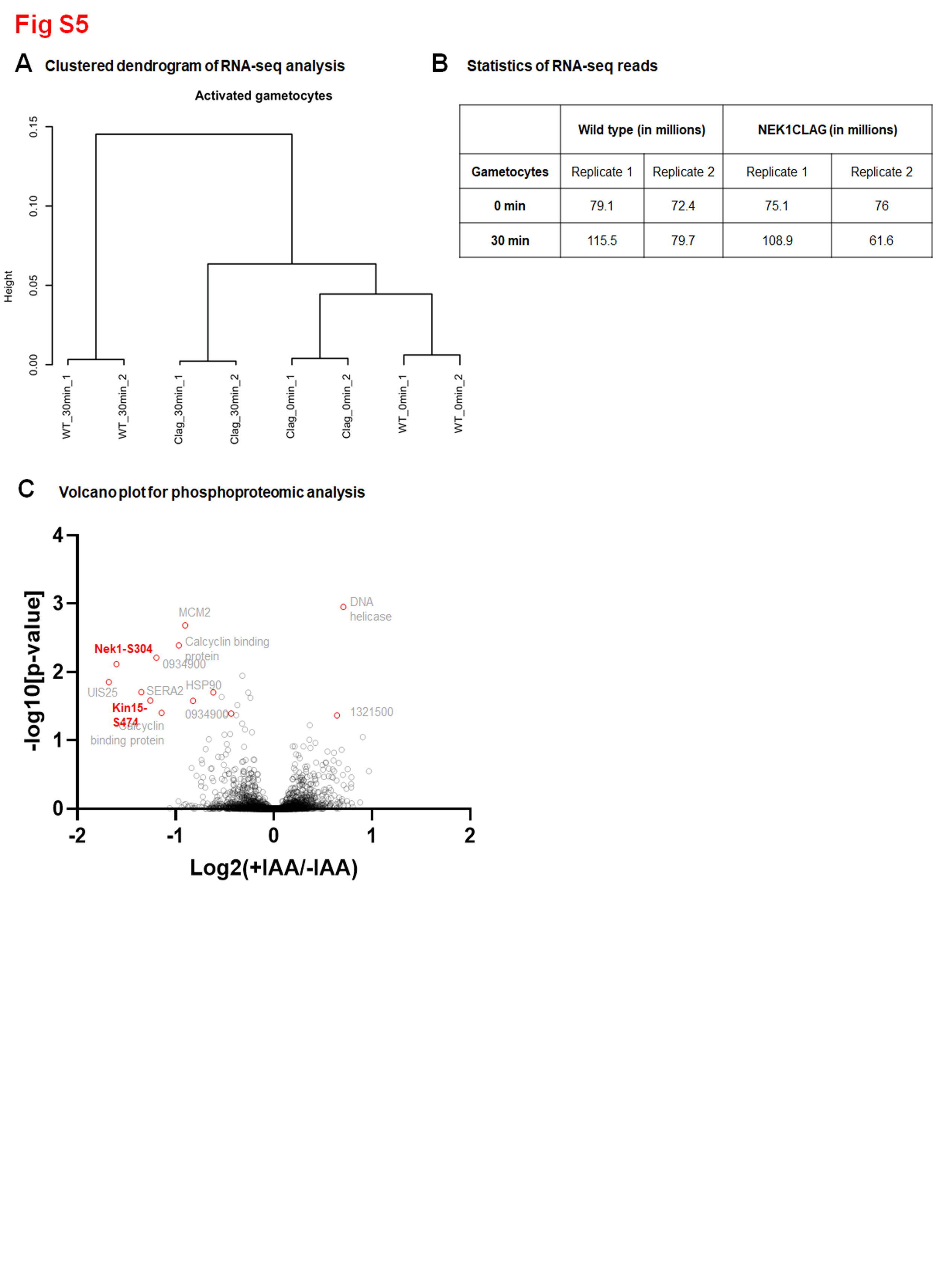

Supplement: S5 Fig — (A) Clustered dendrogram of 2 biological replicates of WTGFP and NEK1clag mutant parasite lines during gametocyte stage (0 min and 30 min) using hierarchical clustering algorithm. Analysis was performed on normalized count data. (B) Table showing RNA-seq read statistics. (C) Volcano plots showing the extent of differentially phosphorylated peptides in gametocytes, comparing NEK1-AID parasites before and after adding IAA. The data underlying this figure can be found in S3 Table. (TIF) [file pbio.3002802.s005.tif]

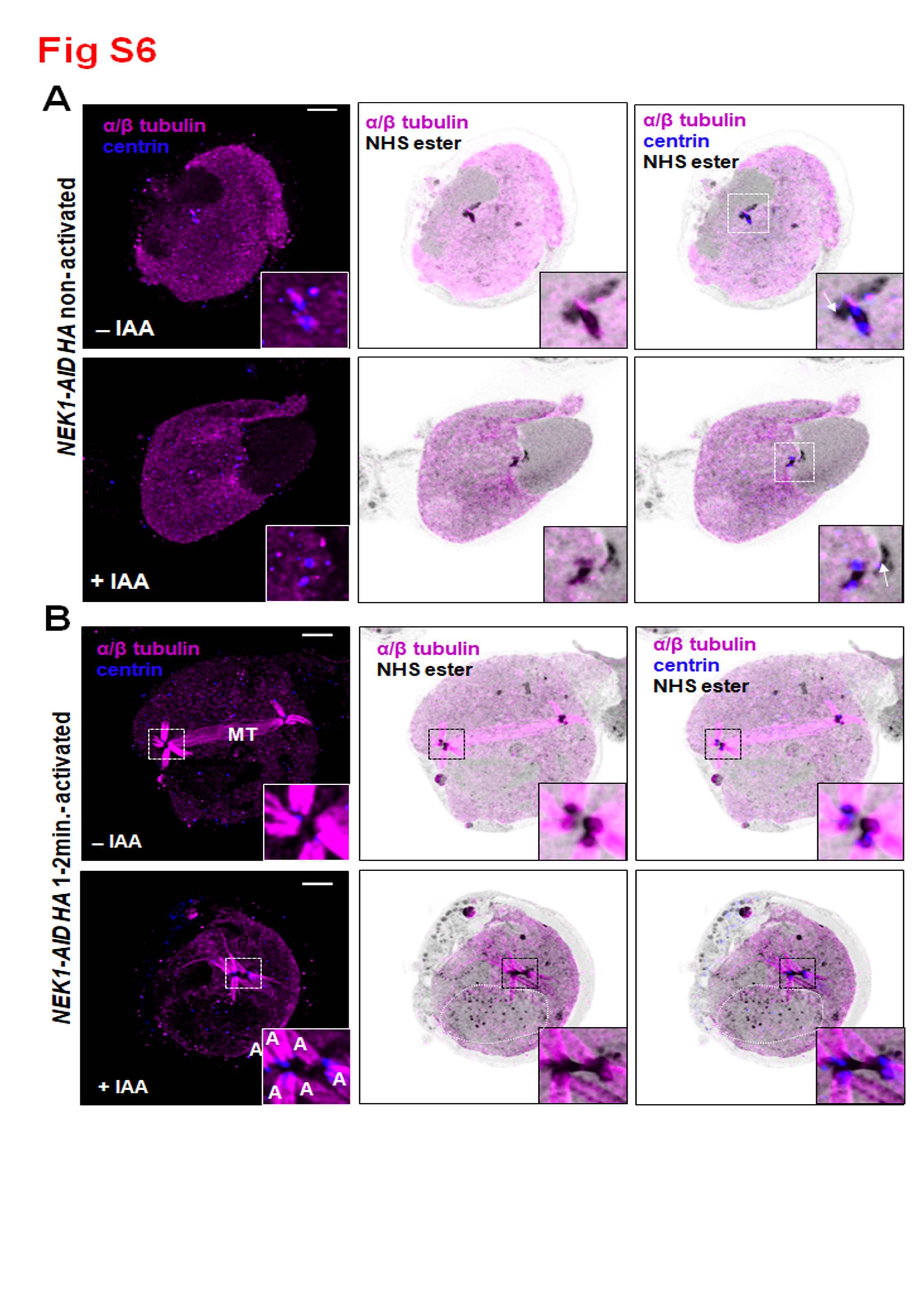

Supplement: S6 Fig — (A) U-ExM images of nonactivated gametocytes with and without depletion of NEK1-AID upon auxin treatment showing identical features highlighting α/β-Tubulin: magenta, centrin: blue, amine groups/NHS-ester reactive: shades of grey, white arrow: intra nuclear body, white insets represent the zoomed area shown around the basal body and MTOC. Scale bars = 5 μm. (B) Depletion of NEK1-AID upon auxin treatment blocks mitotic spindle formation as observed by U-ExM. α/β-Tubulin: magenta, centrin: blue, amine groups/NHS-ester reactive: shades of grey. Axonemes: A; cMTOC: centriolar MTOC; aMTOC: acentriolar MTOC; spindle microtubules: MT. Insets represent the zoomed area shown around centriolar and acentriolar MTOC highlighted by NHS-ester and/or centrin staining. The additional NHS-ester punctate staining in the nucleus most likely correspond to detached kinetochores (dotted white line marked area). Scale bars = 5 μm. (TIF) [file pbio.3002802.s006.tif]

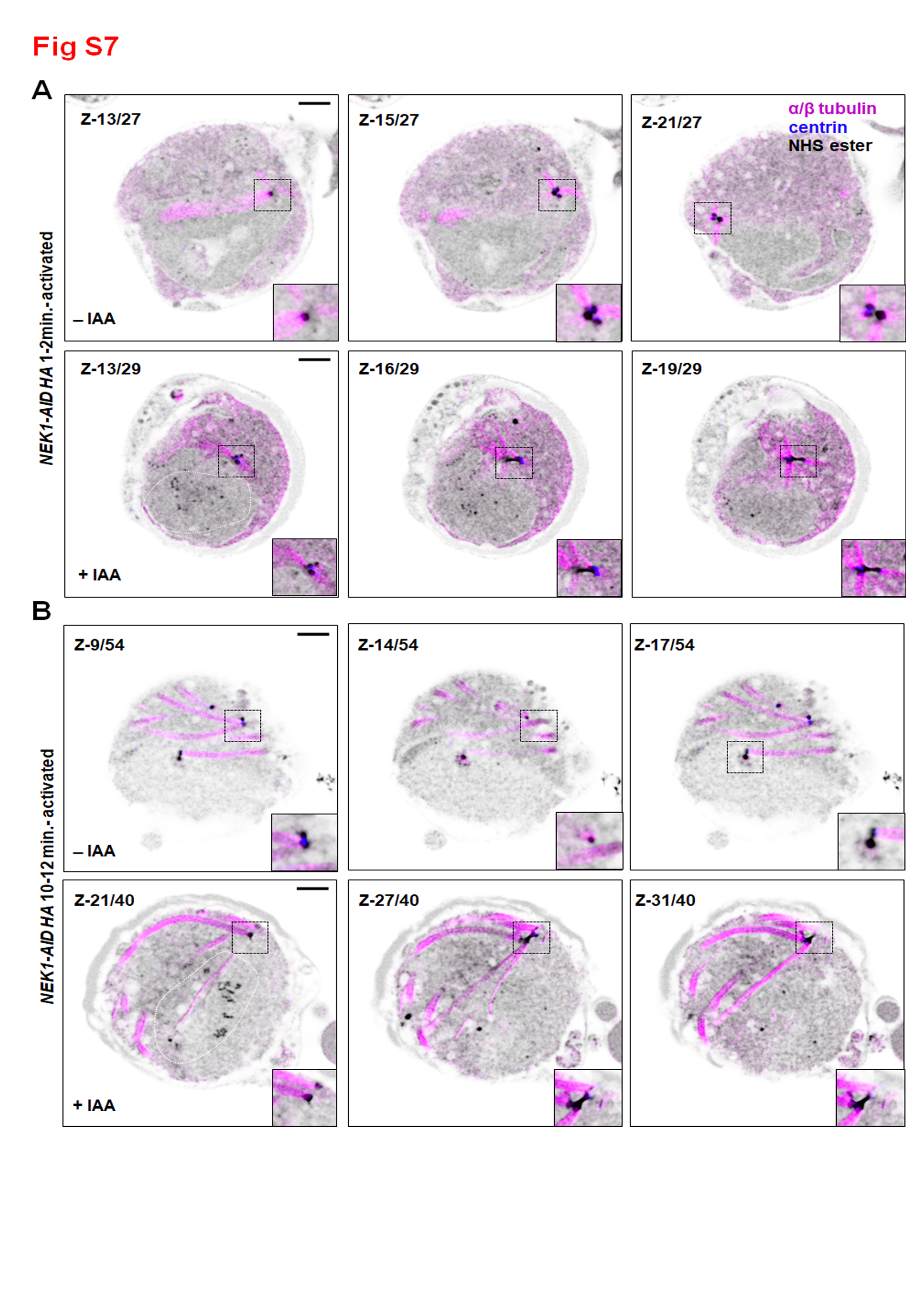

Supplement: S7 Fig — (A) Depletion of NEK1-AID upon auxin treatment blocks mitotic spindle formation as observed by U-ExM in different Z stacks. α/β-Tubulin: magenta, centrin: blue, amine groups/NHS-ester reactive: shades of grey. Axonemes: A; cMTOC: centriolar MTOC; aMTOC: acentriolar MTOC; spindle microtubules: MT. Insets represent the zoomed area shown around centriolar and acentriolar MTOC highlighted by NHS-ester and/or centrin staining. The additional NHS-ester punctate staining in the nucleus most likely correspond to detached kinetochores. Scale bars = 5 μm. (B) Depletion of NEK1-AID upon auxin treatment blocks MTOC segregation as observed by U-ExM. α/β-Tubulin: magenta, centrin: blue, amine groups/NHS-ester reactive: shades of grey. Axonemes: A; cMTOC: centriolar MTOC; aMTOC: acentriolar MTOC; spindle microtubules: MT. Insets represent the zoomed area shown around centriolar and acentriolar MTOC highlighted by NHS-ester and/or centrin staining. The additional NHS-ester punctate staining in the nucleus most likely corresponds to detached kinetochores (dotted white line marked area). (TIF) [file pbio.3002802.s007.tif]
