# Supplementary material for: Plasmodium NEK1 coordinates MTOC organisation and kinetochore attachment during rapid mitosis in male gamete formation
Source: PLoS Biol. 2024 Sep 10;22(9):e3002802. doi: 10.1371/journal.pbio.3002802 (PMC11414920; doi:10.1371/journal.pbio.3002802)

**Raw images used in the manuscript**

**Agarose gel shown in Fig S1B**

Agarose gel showing the PCR product showing correct integration of GFP at C-terminal of NEK1


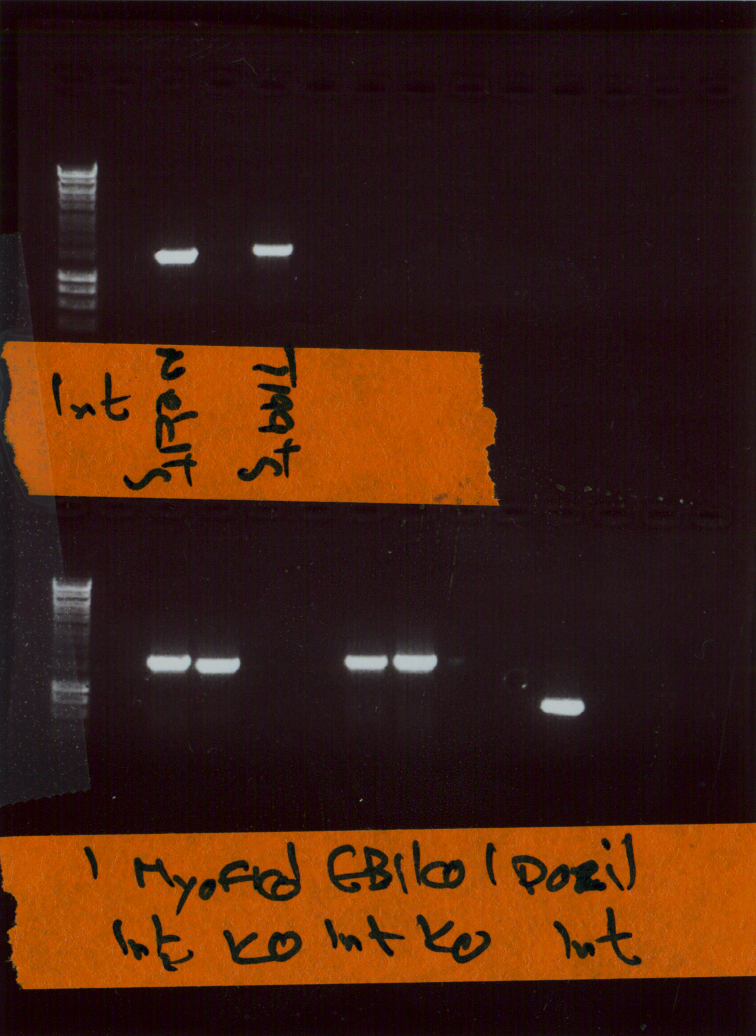


NEK1-GFP

WT

**Raw images-Western blot shown in Fig S1C**

Western blot showing the expression of NEK1-GFP in gametocyte lysate

NEK1-GFP


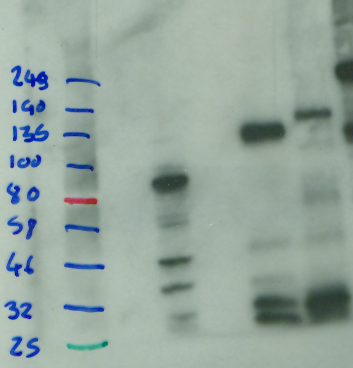
**Raw images-Agarose gel shown in Fig S4B**

Agarose gel showing the PCR product showing correct integration of NEK1-AID


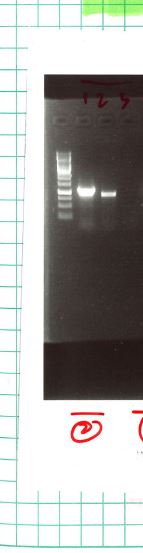


**Raw images-Western blot shown in Fig S4C**

Western blot showing the depletion of NEK1-AID in gametocyte lysate


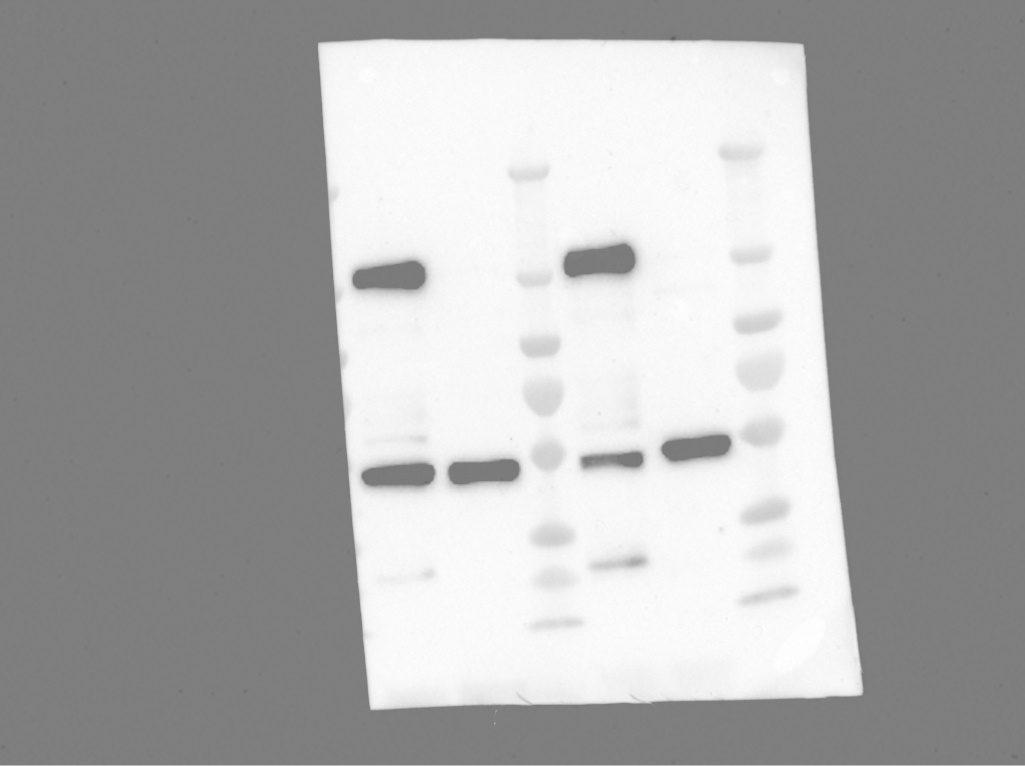


**Raw images-Agarose gel shown in Fig S4F**

Agarose gel displaying the PCR products showing correct integration of NEK1Clag


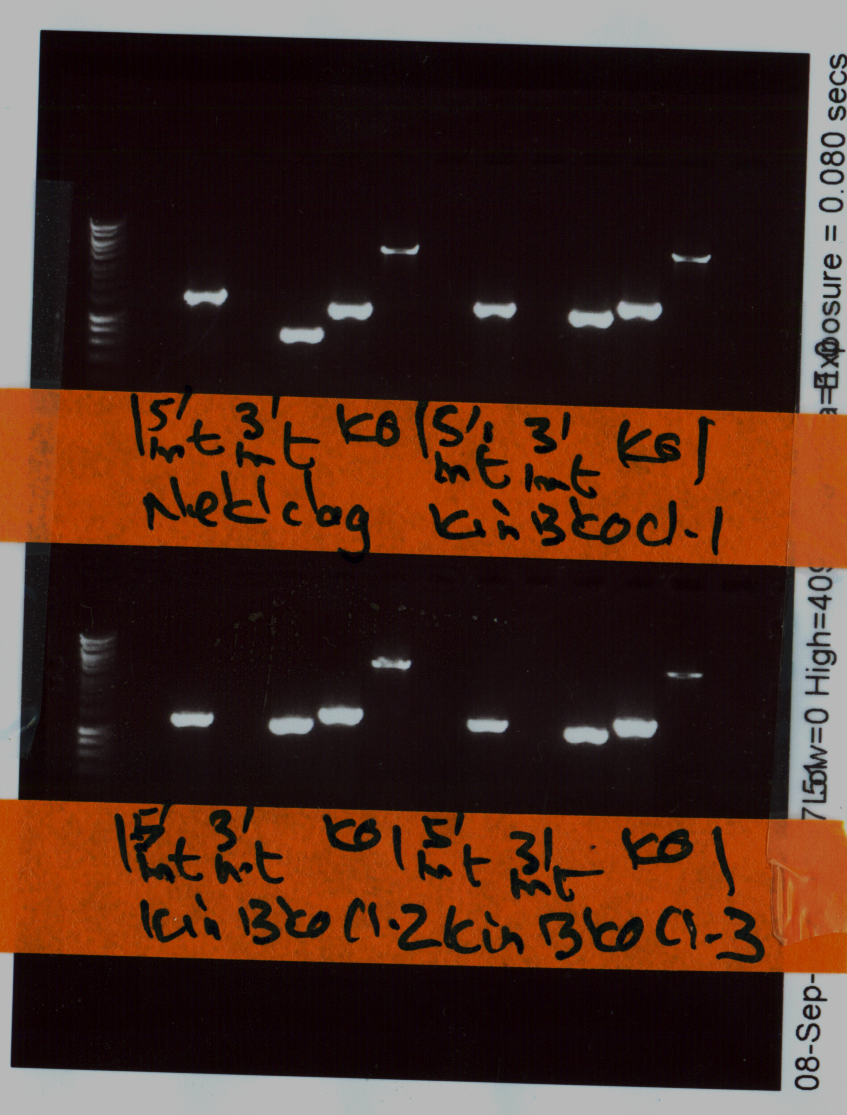

Supplement: S1 Raw Images — (DOCX) [file pbio.3002802.s019.docx]
