# Supplementary material for: Plasmodium NEK1 coordinates MTOC organisation and kinetochore attachment during rapid mitosis in male gamete formation
Source: PLoS Biol. 2024 Sep 10;22(9):e3002802. doi: 10.1371/journal.pbio.3002802 (PMC11414920; doi:10.1371/journal.pbio.3002802)
Supplement: Attachment — Submitted filename: 144294_1_rebuttal_3011397_scwqw3.docx [file pbio.3002802.s020.docx]

**Manuscript number:** RC-2024-02387

**Corresponding author(s):** Rita Tewari

## 1. General Statements [optional]

We wish to thank the reviewers and the Editor for their constructive comments and valuable suggestions to improve our manuscript. We have addressed as far as possible all comments and concerns and we hope that this revised manuscript, with additional new data, will be acceptable for publication. Please find below detailed responses (in red text) to all specific points raised by the reviewers*.*

## 2. Point-by-point description of the revisions

**Response to Reviewers’ comments and suggestions**

We are thankful to the reviewers for their time and effort to review our study and for their constructive suggestions. We address below their comments to further improve the manuscript.

**Reviewer #1**

Major revision points:

The authors should consider using CENH3 as a marker instead of NDC80 to claim NEK1's role in chromosome segregation. Using a direct marker like CENH3 would strengthen their conclusion. However, if the authors choose not to generate the cell line and new set of data, it would be advisable to tone down their conclusion regarding chromosome segregation. I acknowledge the extensive work and data present in the paper.

Response: Thanks to the reviewer for the suggestion. We have tried previously to generate a CENH3-GFP marker line but were not successful. We also requested a CenH3 antibody from the group who published it, but without success. These are the reasons why we generated the NDC80 line, which is another kinetochore marker for chromosome segregation. We have characterised the NDC80-GFP parasite line extensively in a previous study using live cell imaging and super-resolution microscopy to follow its spatiotemporal dynamics at different stages of the Plasmodium life cycle including its correlation with the kinetochore (Zeeshan et al, 2020). We showed its binding to the centromeric region of chromosomes by ChIP seq analysis (See the figure below-Zeeshan et al, 2020). In our recent studies we also showed its dynamic location with other spindle markers like EB1 and ARK2 (Zeeshan et al, 2023). Based on these data, we believe that both CENH3 and NDC80 are appropriate markers for chromosome segregation in Plasmodium, and we hope that the reviewer appreciates this interpretation.

We are pleased to read that the reviewer recognises the extensive amount of work and data present in the paper.

Minor revision points:
The figures are well-designed and presented to a high standard. I especially appreciate the guide schematics associated with the IFAs. However, one area that could be improved is the presentation of the expanded parasites. Firstly, the insets cover a major section of the cell, concealing data from the figure. Secondly, the NHS-ester signal is currently saturated and could be dimmed to more accurately represent the MTOC.

Response: We thank the reviewer for their appreciation and suggestions to further improve the figures. We have shifted the insets on the figures to avoid concealing the data. We have tried to improve the NHS-ester signal and provided more Z-stacks to show the MTOC more accurately. Please see the new supplementary figure S7.

**Reviewer #2 (Evidence, reproducibility and clarity (Required)):**


Major points (please note that both points do not necessarily need further experiments):

1) there is a scarcity for n numbers for many parts of the manuscript. Please give some indications how many cells were inspected, how often a phenotype was observed and how often an experiment was independently carried out (see also minor points).

Response: We have now provided better quantification for all the observations, with the number of cells observed and how many times each experiment was performed reported in the figure legends and in the method section.

2) While the -omics data is interesting, it is somewhat disconnected from the remainder of the manuscript in that it does not lead to any experimental work on the cell level to bring it in connection with NEK1 nor is there any validation. For instance, while the interactome analysis includes a good proportion of very plausible hits, not a single one is validated. I do see that it may be beyond this manuscript to do extensive validations but as this data has limits what can be concluded from it, this should at least be stated in the text. For instance in the paragraph in the discussion (line 613etc). Some experimental validations, if only to show location at the expected site, would be even better.

Response: We thank the reviewer for this insight. We have reanalysed the proteomics data and identified some other MTOC and spindle proteins, including kinesin-8B and kinesin-13 (see new Figure 4). We have already validated kinesin-8B as an MTOC/basal body marker in a previous study, with a similar phenotype during male gametogenesis resulting from its ablation (Zeeshan et al, 2019). Here we show the relative location of NEK1 with respect to that of kinesin-8B, which has a similar MTOC location in early-stage male gametogenesis. In another study we showed that kinesin-13 is localised at the MTOC and spindle and has a similar role during male gametogenesis (Zeeshan et al, 2022). Other proteins, including PF16 and kinesin15, have also been shown to be important for male gametogenesis (Straschil et al, 2010, Zeeshan et al, 2022).

Minor points:
1) Plasmodium is a species name, better not use it on its own (Anopheles would also not be used on its own if Anopheles mosquitoes are meant).

Response: We now mentioned the particular species of the *Plasmodium* genus or in general, we use *Plasmodium spp*.

2) While the imaging is very nice, I do have some issues with backgrounds containing mostly pixels of zero intensity (which seems to be the case for some of the images). In western blots this is not permitted anymore (because it is unclear what was clipped and whether this made weaker bands disappear). The same should apply to microscopy, unless very specific and defined analyses were used that caused this. If this was the case here (e.g. deconvolution, back ground subtraction etc), this should be stated for each type of imaging (including parameters). If this was just due to adjusting levels in Photoshp to clip low intensity, I would recommend to reduce that to a degree where no pixel has 0 intensity anymore to ensure no information in the image was lost.

Response: We thank the reviewer for pointing out this issue. Here, we have used several different types of imaging, including live cell, structural illumination microscopy (SIM), expansion microscopy and fixed immunofluorescence. In general, we adjusted the backgrounds for most of these images as necessary but did not use photoshop for any of them. For live cell imaging, the GFP/RFP fluorescent cell signals are well captured at different time points with an auto-exposure time and then processed simply using Axiovision/Zeiss zen software. SIM images were captured using different parameters based on fluorescence signal intensity and processed to remove background at a threshold level. We provide the processing parameters in the SIM processing method section). For expansion microscopy (ExM), the Z stacks were collected for different channels and the brightness adjusted to remove background. The SIM and ExM resolution images are presented as maximum intensity projections, as explained in the methods section; please see pages 23, 25 and 27. An example of SIM image processing is shown below:

3) Line 158: The role of NEK1 in centrosome splitting in Toxoplasma. Given that Plasmodium NEKs are not analogous to mammalian NEKs, a quick word on the relation of Plasmodium and Toxoplasma NEKs may be beneficial. Does Toxo also have 4 NEKs and is TgNEK1 an orthologoue of Plasmodium NEK1?

Response: In fact, *Toxoplasma gondii* encodes 4 NEKs (Miranda-Saavedra et al., 2012; PMID: 22587893), with TgNEK1 the orthologue of *Plasmodium spp* NEK1.

4) Fig. S1A: is hDHFR really fused to NEK1? Maybe the scheme can be updated to clarify this without readers having to consult the cited publication (Guttery et al., 2014a)

Response: We apologise for the confusion; we have updated and clarified the schematic for readers.

5) Line 135: those other model eukaryotes -> insert "of" before other

Response: We thank the reviewer for spotting this error and have now inserted “of”.

6) Line 149: is substituted by an SMASH -> by a SMASH

Response: We thank the reviewer for spotting this error and have now substituted by “a SMASH”.

7) Line 151: in the modulation of MAPK pathway -> an article seems to be missing here

Response: We thank the reviewer for pointing out the missing article and have now added Dorin-Semblat et al., 2007 article on MAP kinases to the text.

8) Line 153: remove first bracket

Response: We thank the reviewer for noticing the surplus bracket which has now been removed.

9) Line 189; insert „the" before parasite life cycle

Response: We thank the reviewer for noticing this and have now inserted “the”.

10) Line 211: We observed an overlap of NEK1 and centrin signals but in this case NEK1 was closer than centrin to the DNA (Fig 1C). In contrast to the NDC80 statement ("NDC80-mCherry was always closer to the DNA"), there is no quantitative information. Looking at the images this also seems a bit less clear cut. Can the authors put a number to his in some way?

Response: We thank the reviewer for highlighting this issue. To clarify, we have added some images showing the locations of Centrin-4/NDC80/DNA (New Fig 1B). We also calculated the overlap of DNA with centrin and DNA with NEK1 in the images showing the signals of these proteins (new Fig 1E). Similarly, the overlap of DNA with Ndc80 and DNA with NEK1 was calculated in the images showing the signals of these proteins (new Fig 1F). This analysis describes the order of signals for the different markers showing centrin is further than NDC80 from the DNA.

11) Line 216: The live cell images of the proliferative liver schizogony (Fig S1D) and sporogony (Fig S1E) stages showed similar patterns of NEK1-GFP foci formation during proliferative stages. Can the authors specify what they mean with similar? I do not see any change from cytoplasmic to one focus per nucleus in the liver schizont and also in sporogony there only seems one focus per nucleus in the sporozoite.

Response: The focal points are not very clear in these stages. Plasmodium liver and oocyst stages contain thousands of progenies and accurate study of the temporal dynamics of GFP expression is difficult. We highlight here that NEK1-GFP is localised at focal points in the nucleus, together with a diffused cytoplasmic location similar to that in asexual blood and male gametocyte stages. These foci are only observed during proliferative stages in cells undergoing active endomitosis in both oocyst and liver stages. No signal is observed in later stages when nuclear division is finished.

12) Not all videos seem to have been treated the same way. Video 1 shows strong increase in the DAPI signal, suggesting post-acquisition boosting of the signal in later time points to compensate for GFP bleaching. In contrast the DAPI in Video S2 stays in a reasonable dynamic range. Can the type of processing used be indicated in the materials or legends?

Response: We agree with the reviewer. Our main aim here was to observe the dynamic location of GFP- and RFP-tagged proteins at various stages during male gametogenesis, and not focus on quantifying the signal. We adjusted different channels according to fluorescence intensity only to show the protein location. We could collect a series of timelapse images for only two to three minutes because the GFP/RFP signals are bleached quickly.

13) Particularly for Fig. 2F and Fig. S2, Fig. 3I-L and Fig. 6A but also for others, please indicate how many cells and independent experiments this is based on and give the number in the legend (image representative of X inspected cells or something along these lines). Fig. 6B has some of that information in the main text but also their total number of cells inspected should be added to the figure legend.

Response: We thank the reviewer for this important suggestion. We now include the total number of cells analysed for each representative image, and the number of experiments performed, in both the figure legends and methods section.

14) Line318/Figure 3: "Live cell imaging showed that both NEK1 and kinesin-8B were located in the cytoplasm (Fig 3G, S3D)". And also later in this paragraph: Fig. S3D should be S3G and S3H. Also, the recruitment of Kinesin-8B within the first 30 seconds that is mentioned is not shown, a pre-induction image would be generally good to show. At the start of video 6 Kinesin-8B is also already recruited.

Response: We thank the reviewer for this suggestion. We have now included pre-induction gametocytes images showing expression of these proteins in new Fig 3A.

15) Line 339: "the" before nucleus might make this long sentence clearer.

Response: We thank the reviewer for pointing this out. “the” has now been added.

16) Line 341: Fig. 3I, beaded NDC80 signal. This signal does not seem that much different to some of the other markers in the SIM images in this figure part and the signal looks quite processed. How sure are the authors that the beads are real? This would be a very fascinating data point, so maybe worth providing some more image data. Does the number of NDC80 foci make sense? See also point 13.

Response: We thank the reviewer for pointing out this observation. We agree that that the NDC80 signal in this image does not look disimilar from some others, but this beaded structure is present in many images we have captured. Our focus was to locate the NEK1 signal relative to the signal of NDC80, and it was very challenging to capture both focal points, especially using two markers. We have replaced the image in Fig 3I with another with better signals for NDC80 and included more images in supplementary figures (S3I and J) to validate the observation. In previous studies we have observed similar NDC80 foci (about 28 NDC80 foci in a diploid gametocyte). (Zeeshan et al 2022 and Zeeshan et al 2023); please see the following figure. The NDC80 focal points represent the number of unclustered kinetochores; for example, in a diploid gametocyte during spindle formation, this would be expected for the Plasmodium haploid genome consisting of 14 chromosomes and the centromeric region of each chromosome associated with the kinetochore multi-protein complex that facilitates spindle attachment.

17) Is S4A a replicate of Fig. 5A, it looks like the identical gel? Was this done more than once? Maybe also add that it was a 1 h induction time into the figure.
Three replicates were done for the qPCR for the clag promoter strategy, but again this graph is in both, Fig. 5 and Fig. S4. Can the authors weed out the redundancies in these two figures and provide all n numbers?

Response: We thank the reviewer for highlighting this duplication of the image to show depletion/downregulation of NEK1. The image that was originally part of main Figure 5 has now been deleted, leaving it in supplementary Figure S4.

18) Line 419: please help the reader here and modify to start of this paragraph with something along the lines of "To generate PTD lines..."

Response: We have modified the start of the sentence to make clear to readers the importance of the PTD lines.

19) Line 432: Is this how this is typically phrased (In mosquitoes fed NEK1clag parasites)? I would remove "s" from mosquitoes or insert "with" after "fed" (or maybe fed on NEK1clag infected mice?).

Response: We thank the reviewer for this suggestion. We have added “with” after “fed”.

20) Line 436: on the naïve mice, remove "the"

Response: We thank the reviewer for noticing this. We have removed “the” before naïve mice.

21) Line 446: log2fold <1.5 likely should be log2fold>-1.5

Response: We thank the reviewer for pointing this out and have corrected this.

22) Fig. S5: It might be beneficial to give in the figure the information to at a glance see what kind of data the figure parts show because it is a mix of RNASeq, phosphoproteomics, NEK1clag and NEK1-AID/HA gametocytes.

Response: We thank the reviewer for this suggestion and have now described the data/plots showing RNA-seq and phosphoproteomic analyses in Figure S5.

23) Line 500: consider replacing imaging with information.

Response: We thank the reviewer for this suggestion and have now replaced “imaging” with “information”.

24) Line 585: remove "be"

Response: We thank the reviewer for spotting this and have now removed “be”.

25) Not all symbol fonts did survive PDF conversion, see e.g. line 1050 or 1101 or 1197.

Response: We are not quite sure why the symbols did not survive conversion to pdf; we have tested conversion of the Word file to pdf format and all the symbols survived.

Reviewer #2 (Significance (Required)):

The strength of this work is the very comprehensive imaging data that combines several high-end techniques and provides a coherent picture. It has all the necessary markers to firstly localize NEK1 in the context of mitosis and then understand the phenotype when it is inactivated. The weakness of this work lies in the limited quantitative information on some of the observed phenotypes.

Response: We appreciate the concern of the reviewer and have revised the manuscript by adding further quantitative information in figure legends and in the method section.

and in the limited pursuit of the findings of the -omics data although, in favour of the paper - these data do add meaningfully to the overall picture even if they were not further validated.

Response: We have discussed more about the omics data. Please line number 656-58 in discussion

**Reviewer #3**


Major comments:

1. NEK1 mutant parasites show a defect in male gametogenesis. Did authors observe any defect on female gamete formation? This data can be included in the main manuscript. An IFA with female gametocyte marker such as Pbg337 can be included to demonstrate sex-specific expression of NEK1.

Response: We have investigated sex-specificity by using P28 antibody (13.1), a reagent which is generally used in *P. berghei* to identify female gametes and zygotes (we do not have a Pbg337-specific reagent and assume that the reviewer is referring to Pf g377). We see no defect in the number of female gametes formed and identified by surface expression of P28 (Fig S4I and J). This observation was supported by the lack of NEK1-GFP expression in female gametocytes at any time point (Fig. S1F). Furthermore, the rescue experiment (now added in Fig 5F) proves that the defect is only in the male and not the female lineage.

2. Authors should include genetic crosses experiments to demonstrate female gametes of NEK1 mutant are fertile. Authors appear to have sex-sterile lines available in their lab and have performed these experiments in their previous studies (PMID: 37704606).

Response: We thank the reviewer for this suggestion. We have now performed the genetic rescue experiment by crossing NEK1Clag parasites with two female deficient lines (*Δnek4* and *Δdozi*) and one male deficient line (*Δhap2*). In three independent set of experiments, we could rescue the defect in NEK1Clag parasites by crossing with female deficient lines, but not by crossing with the male deficient line, by observing ookinete formation. These data are presented in Fig 5F.

3. Proteomic and phosphoproteomic data using mutant NEK1 parasites showed differential phosphorylation of several proteins but authors did not observe same proteins to be differentially phosphorylated in different replicates. Using in vitro experiments involving peptides or recombinant protein fragments, authors should validate and demonstrate some of the substrates to be direct parasite substrates for NEK1. Kinesin-15 can be one good candidate substrate as kinesin-15 is less phosphorylated in NEK1-AID/HA and is enriched in NEK1-GFP Immunoprecipitates. This is very relevant especially since authors observe perturbation of levels for several other kinases in NEK1 knock down parasites.

Response: We appreciate the reviewer’s suggestion to do in vitro biochemical experimentation to validate NEK1 kinase substrates. With respect, this suggestion is well beyond the focus of this study since our aim was to characterise the cell biology of NEK1 function *in vivo,* with a focus on male gametogenesis. Kinesin-15 together with NEK1 was one of the few proteins for which we detected a significant reduction of phosphorylated peptides across all replicates, despite the high sample variability (likely linked to the pre-treatment involving ethanol and/or auxin to degrade the auxin-induced degron). It is therefore highly likely that phosphorylation of Kinesin-15-S454 is dependent on NEK1. We observed no differential phosphorylation of other kinases in the dataset, but we cannot exclude that other kinases or phosphatases are involved in this signalling pathway.

4. NEK1 is known to phosphorylate MAP2 kinase in vitro in P. falciparum. Did authors find MAP2 to be differentially phosphorylated in their dataset? It should be discussed accordingly in the current manuscript.

Response: We observed phosphorylation of serine 301 in MAPK2. At 6 min post-activation we detected a two-fold reduction of the corresponding peptide with a Q-value of 0.0528, which is just above the selected threshold. Given the high replicate variability, it is possible that this serine is phosphorylated by NEK1 upon gametocyte activation, but more targeted analyses would be necessary to test this hypothesis. As this observation is still rather speculative, we preferred to refer to it in the discussion.

5. Authors show that NEK1 is expressed in pre-erythrocytic stages. Since authors have multiple tools/ transgenic parasites to study relative expression of NEK1, authors can test relative expression of NEK1 in pre-erythrocytic stages and discuss possible function of NEK1 during liver stages.

Response: We appreciate this suggestion of the reviewer, but we consider the proposed work to be outside the scope of this manuscript, where our focus is on the role of NEK1 in sexual cells. We agree that it will be interesting to examine the possible function of NEK1 in liver stages and believe that several groups are now embarking upon such work.


Minor comments:
Authors should cite relevant literature on role of kinases in male gametogenesis by adding a paragraph. e.g. PMID: 18532880, PMID: 29042501, PMID: 29311293, PMID: 32568069, PMID: 34724830, PMID: 32681115, PMID: 36154191 and other kinases.

Response: These references have been added in the discussion section, line numbers 632-635.

Reviewer #3 (Significance (Required)):

Expansion microscopy and live cell imaging are cutting edge and provide the detail and dynamic picture of NEK1 expression in the context of components associated with rapid mitosis, spindle formation, and Kinetochore attachment. This study adds new information to our understanding of the process of male gametogenesis in apicomplexan parasites.

Response: We appreciate these encouraging comments.
