# Supplementary material for: Plasmodium NEK1 coordinates MTOC organisation and kinetochore attachment during rapid mitosis in male gamete formation
Source: PLoS Biol. 2024 Sep 10;22(9):e3002802. doi: 10.1371/journal.pbio.3002802 (PMC11414920; doi:10.1371/journal.pbio.3002802)
Supplement: Attachment — Submitted filename: Responses to comments -15-07-24.docx [file pbio.3002802.s022.docx]

RESPONSES TO ADDITIONAL COMMENTS FROM THE ACADEMIC EDITOR

The study is a thorough analysis of the cell biology of the nek1 kinase during the rapid rounds of mitosis preceding exflagellation of male microgametes. It combines high quality light and electron microscopy to detail the development of centriolar plaques and spindles and the relative positions of various components including NEK1. It combines this with inducible knockdown and promoter trap to prove essentiality of NEK1 for mitosis in male gamete development and it also provides some NEK1 deficient transcriptome & phosphoproteome and NEK1 interactome data. This is a very useful analysis of an enigmatic stage in parasite development that will be valuable not only for the characterisation of NEK1 in this process but as a general reference for the process itself.

We appreciate the Editor’s comment and their recognition of the importance of our study.

I don’t believe any additional experiments are required but some of the analyses needed statistical support or re-writing and some points need clarifying.

I found the results relating to relative positions of nek1 and centrin unconvincing. In asexual blood stage schizonts the supposed difference in dna overlap of NEK1 compared to centrin requires a statistical test to prove there is a difference between correlations for overlaps and might be better tested comparing distance between peak fluorescence intensities rather than correlations of overlap, I have provided more details in my comment on the comment from reviewer 2. In male gametes fig 2e timing of division of centrin and nek1 is convincing but their relative orientation to cytoplasm and nucleus looks like it could just be chromatic shift with both centrin 4 spots shifted in the same direction. From the uEXM in fig 2f the nek1 and centriolar MTOC associated centrin are similar distances from dna at 1 min but by 10 min the nek1 is more nuclear and is dissociating through the nucleus. I think this EXM data for nek1 localisation relative to centrin and dna in male gametes is
much more convincing than the live microscopy and the claims of relative proximity to dna from the live microscopy in fig 2E are confusing and could be deleted.

Response: We agree with the reviewer’s concern about the relative location of centrin and NEK1. By live cell imaging, we observed a similar localization but with NEK1 often closer to the DNA than centrin. In the asexual blood stages, we measured the overlap and corelation coefficient of NEK1 and centrin relative to DNA and this analysis supports the notion that NEK1 is closer to the DNA, though the differences in correlation coefficient were statistically insignificant. Now we have also analysed these data in another way by calculating the distance between the fluorescence peaks of DNA, NEK1 and Centrin/NDC80 signals. Please see (new fig 1E and F). This analysis also supports that NEK1 is localised between NDC80 and Centrin (new fig 1G).

The mitotic process during male gametogenesis is faster (three rounds of mitosis complete in 8-10 min) than in the asexual blood stage, and to capture the orientation of these markers relative to DNA is even more challenging. Therefore, we have provided more images to show their respective locations (Fig S2A). To further support our interpretation of their locations and orientation, we used UExM to show that NEK1 is located closer to the DNA (shown in fig 2F). Overall, we show that NEK1 shows co-localization with centrin (MTOC) and that centrin splits first during mitosis.

Fig 4, mass spec, is missing and there is no fig 5j, please carefully check fig numbering in this revised MS

Response: Mass spec data is provided in supplementary table S1.

We are sorry for the confusion about figure 5 numbering, particularly regarding Fig 5J. We have corrected the figure numberings.

Line 456 RNASeq, I suggest rephrasing “all replicates were clustered together”, this is a bit ambiguous and could be interpreted to mean that duplicates were pooled prior to diff gene expression analysis when the authors actually mean the replicates were reproducible and similar to each other in a clustering analysis, maybe just say the replicates were reproducible?

Response: Thanks for this suggestion. We have modified the sentence accordingly in line number 479.

I believe that the transcriptome and proteomes are useful information but also that these analyses are difficult to interpret when they compare parasites that have had interrupted development, particularly for the 30 min post activation rnaseq. Would 30 min activated rnaseq be somewhat confounded by the reduced number of male compared to female gametes? Are the genomes actively transcribed in microgametes? Could the data be compared to sex specific transcriptomes/proteomes to exclude the differential kinase transcription and the peptide differences being a consequence of no male microgametes being produced rather than a direct consequence of NEK1 phosphorylating a regulator of these genes/proteins? Perhaps just include some discussion of the potential confounders for these analysis when comparing developmentally different parasites.

Response: There were few differences in the transcriptome 30 min post activation due to downregulation of NEK1 compared to wild type. The minor differences suggest that there is no or minimal involvement of NEK1 in modulation of gene transcripts.

The editor is correct to suggest that any difference may be due to the reduced number of males compared to female gametes. In previous studies we have seen differences in the transcriptomes of gametocytes activated for 30 mins upon deletion or downregulation of certain proteins like kinesin-8X (Zeeshan et al, 2019- PMID: 31600347) and protein phosphatase 1 (Zeeshan et al, 2021- PMID: 34145386). As far we are aware the male gamete genome is not actively transcribed and the female gamete is under transcriptional repression until fertilization. We did not analyse the data based on a sex-specific transcriptome.

The phosphoproteomic analysis was done at 1-2 min, 6 min and 12 min post gametocyte activation and did not show any major differences except for one protein, kinesin15. Previous studies have shown that these are important time points to detect differential phosphorylation events (Invergo et al, 2017-PMID: 29141230; Balestra et al, 2020-PMID: 32568069).

These results suggest that downregulation of NEK1 does not modulate the transcriptome, and depletion of NEK1 does not result in detectable differences in the phosphoproteome during gametogenesis. The slight changes in transcriptome could be due to a reduced number of male gametes.

The legend for the symbols within fig 6 needs to be aligned

Response: These have now been realigned.

Re reviewers comments
Reviewer 1
I think NDC80 is an OK surrogate for the centromeres at the stages examined. The authors could include a conditional comment though to make it clear that NDC80 is not an integral component of the centromere but has only a centromeric location in their chipseq. I note though that the chipseq is presumably only a single timepoint so may not be representative of the stages in the figures.

Response: We thank the reviewer for their agreement that NDC80 is a surrogate marker for the centromere. We have now added a sentence to explain the location of NDC80 on the centromere, consistent with the suggested role of NEK1 in chromosome segregation (line no.333).

Reviewer 2
Major points

2) validating omics. I don’t think this is necessary, the authors were describing the function of nek1, they have done a thorough job by identifying candidate interacting factors, they are useful leads for future work but proving them or investigating them further is really beyond the scope of a study focusing on nek1.

Response: We thank the Editor for considering that the omics validation is beyond the scope of this study.

Minor points

2) subtraction of background from imaging.
The reviewer mentions photoshop but I think the program used is irrelevant, ie axiovision might also be used to set a threshold value for background below which everything is zero. This is the same issue raised by reviewer 1. I have not analysed sim or exm myself so I do not know whether maximum intensity projections is acceptable. For widefield or confocal fluorescence microscopy I would not accept the subtraction of "background" below a threshold level as this constitutes removing data. I agree with the reviewers that the presented image should show the full dynamic range of the data with background having a detectable value and maximum signal not being saturating.

Response: We have revisited all the images and processed some of them again keeping the threshold level of background as having a detectable value and the maximum signal not being saturating. Please see the modified figures 2, 3, and supplementary fig S3. We hope that this improvement addresses these concerns.

10) fig1c, comparison of nek1 centrin distance to dna, quantitation requested.
For figs 1E and F there are more than 30 measures for each plot so the authors should perform a statistical test to support their claim that NEK1 is closer than centrin to dna and ndc is closer than nek1 to dna. Actually I think that correlation is perhaps not the best measure to compare different proteins if the compared measure is "distance from". If a line were drawn through peak fluorescence it is not clear to me from the supplied images that peak centrin would be any further from dna than would nek1.

Response: We have performed a statistical test, and this is mentioned in the methods section of live cell imaging (last paragraph) and in figure legends of new Fig. 1E, F and supplementary Fig 1E and F . We have moved the overlap analysis into Supplementary Fig 1. We have now analysed the distance between the intensity peaks to make the analysis clearer (please see new Fig. 1E, F)

11) similarity of nek1 in liver stages.
I accept the authors’ explanation but they have not amended the original text to clarify this, which they should, because it was not clear to the reviewer and so is also likely to be unclear to multiple readers. I suggest they add the adjective nuclear before the noun foci.

Response: Thanks for pointing out this missing information. We have now amended the text to clarify this, and we have also added ‘nuclear’ before ‘foci’ (please see lines 229-235).

12) video dapi signal saturation.
The authors explain why they saturated dapi but they need to clarify what they have done to alter MS as per the reviewers request, ie how have material or legends been altered to indicate differences in processing?

Response: We have clarified this issue in the methods section, lines 769-776 and supplementary video legend.

14) preinduction image of kinesin 8B.
Fig 3A is labelled ndc80, not kinesin 8B, have the authors supplied the pre-induction image of kinesin 8b as requested and as they have stated?

Response: We apologise for the confusion. We have now provided the preinduction images for all markers including kinesin-8B in Supplementary Fig S3A. Please see lines 313-316.

16) NDC80/kinetochores arrayed along spindle
The explanation in the text seems clear that nek1 is in the mtocs and the ndc80/centromeres are organised along the spindle, I dont think this requires further explanation.
Response: We thank the Editor for this consideration.

Reviewer 3
Major comments

3) validating (phosphor)proteomics
Validating the kinase target is probably beyond the scope of the study, I think the cell biology plus the omics screens is sufficient. The omics analyses are not definitive proof but provide leads for future studies which seems appropriate appropriate to me. I note that another reviewer requested follow-up to the interacting proteome. In light of the multiple omics sets analysed I think following up is beyond the scope of one study.
Response: We appreciate the comment from the Editor that this is beyond the scope of the study.

5) NEK1 in liver stages
I think that analysis of liver stages is beyond the scope of the MS

Response: We thank Editor for this comment.
